# BLISTER Regulates Polycomb-Target Genes, Represses Stress-Regulated Genes and Promotes Stress Responses in *Arabidopsis thaliana*

**DOI:** 10.3389/fpls.2017.01530

**Published:** 2017-09-11

**Authors:** Julia A. Kleinmanns, Nicole Schatlowski, David Heckmann, Daniel Schubert

**Affiliations:** ^1^Plant Developmental Epigenetics, Heinrich Heine Universität Düsseldorf Düsseldorf, Germany; ^2^Computational Cell Biology, Heinrich Heine Universität Düsseldorf Düsseldorf, Germany; ^3^Epigenetics of Plants, Freie Universität Berlin Berlin, Germany

**Keywords:** ABA, abiotic stress, BLISTER, CLF, drought, H3K27me3, polycomb, PRC2

## Abstract

**HIGHLIGHTS**

- The PRC2 interacting protein BLISTER likely acts downstream of PRC2 to silence Polycomb target genes and is a key regulator of specific stress responses in *Arabidopsis*.

Polycomb group (PcG) proteins are key epigenetic regulators of development. The highly conserved Polycomb repressive complex 2 (PRC2) represses thousands of target genes by trimethylating H3K27 (H3K27me3). Plant specific PcG components and functions are largely unknown, however, we previously identified the plant-specific protein BLISTER (BLI) as a PRC2 interactor. BLI regulates PcG target genes and promotes cold stress resistance. To further understand the function of *BLI*, we analyzed the transcriptional profile of *bli-1* mutants. Approximately 40% of the up-regulated genes in *bli* are PcG target genes, however, *bli-1* mutants did not show changes in H3K27me3 levels at all tested genes, indicating that BLI regulates PcG target genes downstream of or in parallel to PRC2. Interestingly, a significant number of BLI regulated H3K27me3 target genes is regulated by the stress hormone absciscic acid (ABA). We further reveal an overrepresentation of genes responding to abiotic stresses such as drought, high salinity, or heat stress among the up-regulated genes in *bli* mutants. Consistently, *bli* mutants showed reduced desiccation stress tolerance. We conclude that the PRC2 associated protein BLI is a key regulator of stress-responsive genes in *Arabidopsis*: it represses ABA-responsive PcG target genes, likely downstream of PRC2, and promotes resistance to several stresses such as cold and drought.

## Introduction

Epigenetic gene regulation has important roles in genome defense but also in the regulation of development and the response of organisms to environmental cues and can be mediated by posttranslational modifications of histones or DNA methylation. Polycomb group proteins are key epigenetic regulators of development, assemble in large complexes and maintain gene repression. The conserved Polycomb Repressive Complex 2 (PRC2) consists of four core members and silences target genes by tri-methylation of histone 3 lysine 27 (H3K27me3). In *Arabidopsis thaliana*, PRC2 is composed of one of three SET domain-containing histone methyltransferases MEDEA (MEA), SWINGER (SWN), and CURLY LEAF (CLF); one of three VEFS domain-containing proteins EMBRYONIC FLOWER 2 (EMF2), FERTILIZATION INDEPENDENT SEED 2 (FIS2), and VERNALIZATION 2 (VRN2); and the two WD40 domain-containing proteins FERTILIZATION INDEPENDENT ENDOSPERM (FIE) and MULTICOPY SUPPRESSOR OF IRA 1 (MSI1) (reviewed in Derkacheva and Hennig, 2014). The loss of PRC2 function leads to a loss of H3K27me3 at PcG target genes which may be associated with ectopic expression of those genes. Trithorax group (TrxG) proteins, such as ARABIDOPSIS TRITHORAX1 (ATX1) (Alvarez-Venegas et al., 2003), ULTRAPETALA1 (ULT1) (Carles and Fletcher, 2009), and BRAHMA (BRM) (Farrona et al., 2004), act antagonistically to PRC2. TrxG proteins activate gene expression through setting H3K4me3 and by ATP-dependent chromatin remodeling.

As plants are sessile organisms, they need to rapidly respond to abiotic and biotic stresses, e.g., by altered gene expression and metabolite production. Plant stress responses that result in osmotic imbalance and cell desiccation, such as drought, high salinity, and cold, partially rely on the phytohormone abscisic acid (ABA). Early in development ABA regulates seed maturation and maintains seed dormancy. During vegetative development ABA is involved in general growth and reproduction and plays an important role in the response to stress (reviewed in Tuteja, 2007). Epigenetic gene regulation likely plays important roles in regulation of stress responses, as stress-responsive genes need to be repressed in non-stress conditions and stress may create a memory for a faster and more pronounced response to a recurrent stress. Previously it was shown that the PRC2 component MSI1 is a negative regulator of drought stress response (Alexandre et al., 2009). Recently a study revealed that MSI1 functions in a histone deacetylase complex to fine-tune ABA signaling and that loss of MSI1 led to an increased tolerance to salt stress (Mehdi et al., 2016). The levels of H3K27me3 were not analyzed in both studies (Alexandre et al., 2009; Mehdi et al., 2016), therefore, it remained unclear whether the PcG associated function of MSI1 is involved in the regulation of stress-responsive genes. Interestingly, loss of CLF results in a reduced resistance to drought (Liu et al., 2014) suggesting that different PRC2 members have distinct functions in regulating stress responses (despite that there is a common ectopic expression of some genes such as LTP3 and LTP4), that PRC2 mutants may display opposite phenotypes due to the level of PRC2 reduction or that the role of MSI1 in drought stress regulation is due to its function in additional complexes.

We previously showed that CLF is interacting with the plant-specific, coiled-coil protein BLISTER (BLI) (Schatlowski et al., 2010) which promotes the resistance to cold stress (Purdy et al., 2010). *BLI* is ubiquitously expressed throughout development and its loss results in a strong pleiotropic phenotype with mutants displaying affected seed, leaf, and flower development and a strong reduction in plant size. We previously showed that BLI regulates expression of several PcG target genes but likely also has PcG-independent functions (Schatlowski et al., 2010), but it remained unclear whether BLI is general regulator of PcG target genes and whether the function of BLI in the cold stress response is linked to PcG function.

Here, using transcriptional profiling of *bli-1* mutants, we revealed that a significant number of PcG target genes is differentially expressed and that a significant number of those genes is regulated by ABA. Importantly, de-repression of PcG target genes in *bli* is not due to reduced H3K27me3 levels, indicating a role of BLI downstream of or in parallel to PRC2 function. Furthermore, we report that in *bli* mutants a high number of stress-responsive genes is differentially expressed and that *bli* mutants display a reduced tolerance to desiccation stress. We propose that BLI is not only involved in the positive regulation of desiccation stress but might function as a general regulator of stress responses which is achieved in part by regulating stress-responsive PcG target genes.

## Materials and methods

### Plant material and growth conditions

Seeds of Columbia-0 (Col-0, N1092), *bli-1* (SAIL_107_D04, N805222), *bli-11* (GABI-Kat_663H12), *clf-28* (SALK_139371, N639371), *bli-1*/BLI:BLI-GFP (Supplemental Materials and Methods), *bli-11*/BLI:BLI-GFP (Supplemental Materials and Methods) were sterilized (10 min 70% Ethanol supplemented with 0.05% Triton X-100, 10 min 96% Ethanol) and sown on ½ MS medium (half-strength Murashige and Skoog medium supplemented with 0.5% sucrose and 0.05% MES). Seeds were stratified for 2 days at 4°C and grown under LD conditions, (8/16 h dark/light rhythm at 20°C). *bli-1* and *bli-11* seeds showed a germination delay of 2 days (Schatlowski et al., 2010), therefore these two genotypes were sown 2 days earlier than all other genotypes when directly compared, stratified for 2 days at 4°C and then transferred to the respective growth condition. For GUS staining plants were grown for 14 days on ½ MS under LD conditions.

### Microarray analysis

Seeds for microarray experiments were sterilized and sown on ½ MS medium. Seeds were stratified for 2 days at 4°C, grown under continuous light conditions for 12 days and then harvested. RNA from whole seedlings was extracted using RNeasy Plant Mini Kit (Qiagen, Hilden), resuspended in 30 μl RNAse-free water and treated with DNase (Fermentas). The quality was determined using a Bioanalyzer eukaryote total RNA nano chip (in cooperation with BMFZ, HHU Düsseldorf). RNA samples were processed by imaGenes GmbH (Berlin) with Agilent technologies using Arabidopsis 44 k single color arrays. The microarray was analyzed using background correction and quantile normalization of the limma package in the R environment (R Core Team, 2015; Ritchie et al., 2015). Differential expression was estimated using the empirical Bayes statistics implemented in limma (Smyth, 2004; Ritchie et al., 2015). *P*-values were adjusted for multiple testing using the Benjamini-Hochberg method (Benjamini and Hochberg, 1995). Genes with a fold-change equal to or higher than 1.5 and with a *p*-value below 0.05, were included in further analyses.

*bli-1* differentially expressed genes were compared to indicated gene sets using VirtualPlant 1.3 (Katari et al., 2010). For GO term analysis we used the online resource GOToolbox (http://genome.crg.es/GOToolBox/), using hypergeometric test with Benjamini-Hochberg correction for statistical analysis and *p*-value determination. For GOSlim analysis we used the online resource at “The Arabidopsis Information Resource” website (http://www.arabidopsis.org/tools/bulk/go/index.jsp) and statistically analyzed the data by Chi square test with Yates correction. Oligonucleotide sequences for validation of expression can be found in Table S2.

Microarray data have been submitted to GEO (GSE100815).

### Chromatin immunoprecipitation (ChIP)

ChIP was performed as previously described (Schatlowski et al., 2010). Briefly, plants were grown for 14 days on ½ MS medium under LD conditions. 0.3–1 mg of seedlings were crosslinked using 1% FA fixation solution (10 mM Tris pH 7.5, 10 mM EDTA, 100 mM NaCl, 0.1% Triton X-100, 1% Formaldehyde) for 20 min under vacuum on ice. 2 M glycine was added to a final concentration of 0.125 M to stop the crosslink reaction. Samples were rinsed with ice-cold water to remove the fixation solution and frozen in liquid nitrogen.

Twenty microliters of Protein A coupled beads (Thermo Fisher Scientific) per sample were washed 3x with ChIP dilution buffer (1.1% Triton X-100, 1.2 mM EDTA, 16.7 mM Tris pH8, 167 mM NaCl, 0.2 mM PEFABLOC), then 1 μg antibody (anti-H3K27me3, C15410195 Diagenode; anti-H3K4me3, C15410003 Diagenode; anti-igG, C15410206 Diagenode) per 20 μl beads were added and incubated rotating 10–12 h at 4°C. Frozen samples were ground in liquid nitrogen to a fine powder. Then 30 ml of Extraction buffer 1 (0.4 M sucrose, 10 mM Tris-HCl pH 8, 10 mM MgCl_2_, 5 mM beta-mercaptoethanol, 0.2 mM PEFABLOC, 1:200 plant proteinase inhibitor cocktail, 1 mM EDTA) were added to the powder, samples were vortexed and incubated 5 min on ice. The solution was filtered twice through 1 layer of miracloth (VWR) and centrifuged for 20 min at 5,000 g at 4°C. Supernatant was removed and pellet was washed twice with 1 ml Extraction buffer 2 (0.25 M sucrose, 10 mM Tris-HCl pH 8, 10 mM MgCl_2_, 1% Triton X-100, 5 mM beta-mercaptoethanol, 0.2 mM PEFABLOC, 1:200 plant proteinase inhibitor cocktail, 1 mM EDTA). Samples were resuspended in 300 μl extraction buffer 3 (1.7 M sucrose, 10 mM Tris-HCl pH 8, 2 mM MgCl_2_, 0.15% Triton X-100, 5 mM beta-mercaptoethanol, 0.2 mM PEFABLOC, 1:200 plant proteinase inhibitor cocktail, 1 mM EDTA) and layered on 300 μl extraction buffer 3 (sucrose gradient), centrifuged for 1 h at 16,000 g at 4°C. The pellet was resuspended in 300 μl Nuclear Lysis Buffer (50 mM Tris-HCl pH 8, 10 mM EDTA, 1% SDS, 0.2 mM PEFABLOC, 1:200 plant proteinase inhibitor cocktail) and samples were sonicated 10–12 × (30 s on, 60 s off). Nuclear debris were removed by centrifugation for 5 min at 12,000 g at 4°C. Antibody-coupled beads and the no-antibody control beads were washed 3x with ChIP dilution buffer. One hundred microliters of sample and 900 μl of ChIP dilution buffer were added to 20 μl of beads and incubated rotating 10–12 h at 4°C for IP. Beads were washed 2x each with low salt wash buffer (150 mM NaCl, 0.1% SDS, 1% Triton X-100, 2 mM EDTA, 20 mM Tris-HCl pH 8), high salt wash buffer (500 mM NaCl, 0.1% SDS, 1% Triton X-100, 2 mM EDTA, 20 mM Tris-HCl pH 8), LiCl wash buffer (0.25 mM LiCl, 1% Nonidet-40, 1% sodium deoxycholate, 1 mM EDTA, 10 mM Tris-HCl pH 8) and 1x with TE buffer (10 mM Tris-HCl pH8, 1 mM EDTA). Elution was achieved by adding 500 μl of 65°C warm elution buffer (1% SDS, 0.1 M NaHCO_3_) to the samples and incubating them for 30 min at 65°C with gentle shaking. The eluate was reverse-crosslinked by adding 20 μl of 5 M NaCl and incubation for 6–12 h at 65°C. Proteins were removed by adding 1 μl Proteinase K (20 mg/ml, Thermo Fisher Scientific), 10 μl 0.5 M EDTA and 20 μl 1 M Tris-HCl (pH 6.5) and incubation for 60 min at 45°C. DNA was recovered using Phenol/Chlorophorm. The DNA pellet was resuspended in 50 μl dH_2_O. For qPCR analysis 2 μl of a 1:10 dilution of the DNA samples were used.

qPCR was performed in a CFX384 Touch Real-Time PCR Detection System (Bio-Rad) using KAPA SYBR FAST qPCR Master Mix in a 2-step PCR program [95°C 3:00 min, 40 × (95°C 0:05 min, 60°C 0:30 min)]. Values for immuno-precipitation (IP) were referred to input samples (= %IP). To account for differences in IP efficiencies and depending on the analyzed modification, %IP values were normalized to the *FUSCA3* locus (AT3G26790), which carries H3K27me3 and is not expressed in wild type and *bli-1*, and *ACTIN7* (AT5G09810), which carries H3K4me3 and is strongly expressed in wild type and *bli-1*. Oligonucleotide sequences can be found in Table S1.

### Principal component analysis

Expression profiles of responses to abiotic stress were obtained from the AtGenExpress dataset (Kilian et al., 2007) and a study on ER-stress induced by the chemical tunicamycin (Nagashima et al., 2011). The dataset of Nagashima et al. (2011) was evaluated using the robust multi-array average (RMA) expression measure (Wu and Irizarry^1^); the AtGenExpress data was provided in preprocessed form. Comparable distributions of gene expression were produced by quantile normalization, and replicates were averaged to compute fold changes. In the cases of stress treatment, expression was normalized against control, while the data on *bli-1* was normalized against wild type. We performed Principal Component Analysis on the log2-tranformed fold changes in gene expression using the prcomp() function of the stat package in R (R Core Team, 2015).

### Stress experiments

For desiccation stress experiments, petri dishes with ½ MS medium in LD conditions were covered with 4 separate membrane pieces (Sefar Nitex membrane 03-200/54, pore size: 200 μm/diameter) and sterile seeds were placed on top of each membrane (for visualization of experimental setup see Figure S4). Seeds were stratified for 2 days at 4°C and grown under LD conditions. The membranes pore size of 200 μm/diameter ensured proper imbibition of seeds and a penetration by roots. Dessication stress was applied 5 days after germination. Under a sterile bench the membranes with young seedlings were transferred to sterile empty petri-dish lids. For the 0 h control, membranes were lifted up and directly placed back on ½ MS plates to avoid possible artifacts caused by lifting up the membrane. Constant airflow in the sterile bench ensured that the seedlings placed on lids were exposed to desiccation. After 0, 0.5, 1, and 2 h the membranes with seedlings were transferred back to the ½ MS plates. After stress treatment, seedlings were grown for additional 5 days on ½ MS plates and survival was scored.

### GUS staining

Detection of ß-Glucuronidase (GUS) activity was performed according to Jefferson et al. (1987) with some modifications. Plants were fixed with 90% acetone for 30 min on ice and then washed for 20 min on ice with solution I [35 mM Na_2_HPO_4_, 13 mM NaH_2_PO_4_, 0.5 mM K_3_Fe(CN)_6_, 0.5 mM K_4_Fe(CN)_6_, 1 mM EDTA, 500 μl Triton X-100 in 50 ml dH_2_O]. Solution I was replaced by GUS-staining solution [35 mM Na_2_HPO_4_, 13 mM NaH_2_PO_4_, 0.5 mM K_3_Fe(CN)_6_, 0.5 mM K_4_Fe(CN)_6_, 500 μl Triton X-100, 5 mg X-Gluc in 50 ml dH_2_O] and samples were incubated for 2–12 h at 37°C. Samples were washed with dH20 and destained with 70% Ethanol. Plants were analyzed and imaged using a stereomicroscope (Stemi 2000-C, Zeiss) equipped with AxioCam ICc1 (Zeiss).

## Results

To further understand whether PcG target genes are overrepresented within the BLI regulated genes, we performed a microarray experiment using a 44 k Agilent array. We used *bli-1* seedlings grown for 12 days under continuous light conditions and compared the transcriptional profile to the Col-0 wild type.

### Transcriptional profiling reveals a functional overlap of BLI and CLF target genes

**Figure 1:**
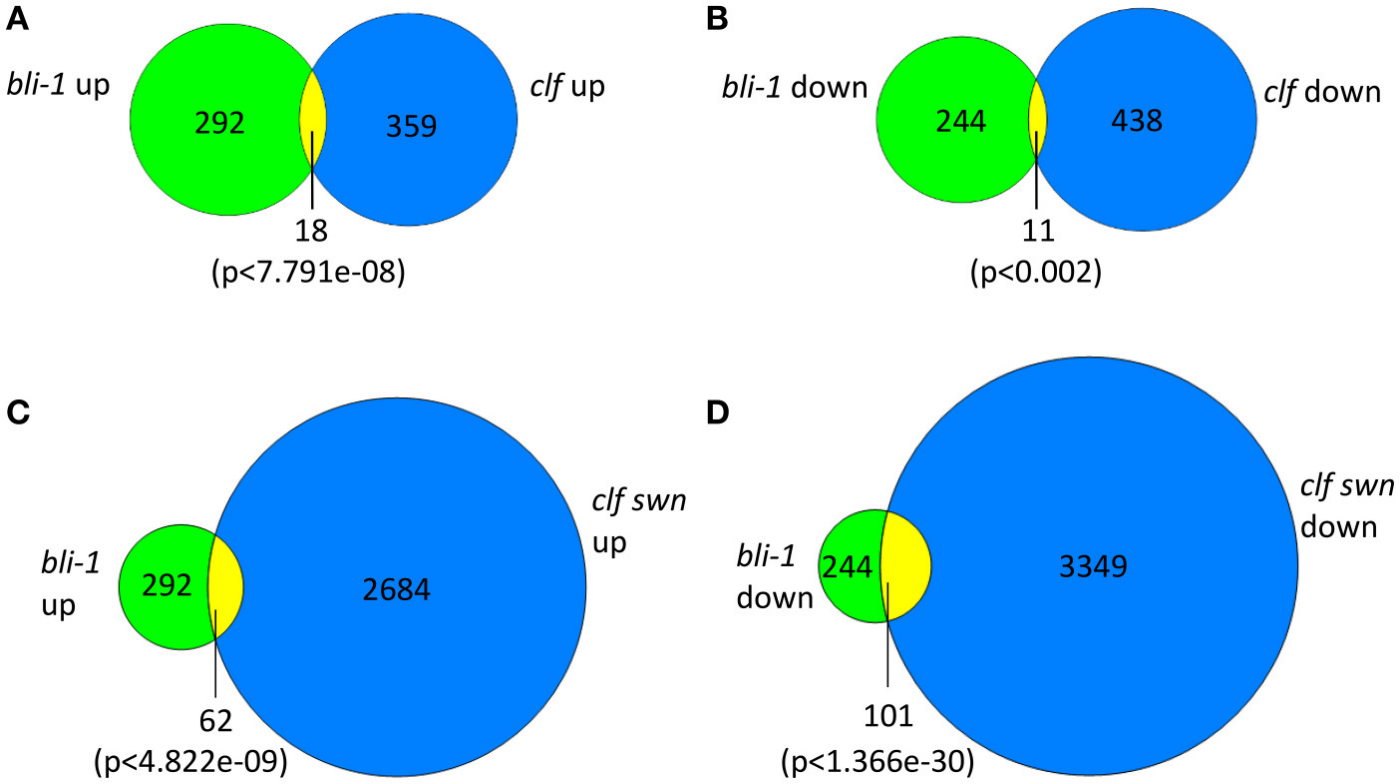
Venn diagrams of differentially expressed genes in *bli-1* compared to *clf-28* and *clf swn* double mutants. **(A)** Comparison of up-regulated genes in *bli-1* seedlings vs. up-regulated genes in *clf-28* (Farrona et al., 2011). **(B)** Comparison of down-regulated genes in *bli-1* seedlings vs. down-regulated genes in *clf-28*. The comparison of differentially expressed genes in *bli-1* and *clf-28* revealed a significant overlap between the two mutants. **(C)** Comparison of *bli-1* and *clf swn* up-regulated genes. **(D)** Comparison of *bli-1* and *clf swn* down-regulated genes. The overlap of *bli-1* and *clf swn* differentially expressed genes was highly significant. Statistical significance was tested using the hypergeometric distribution; a *p*-value below 0.05 was considered as statistically significant.

In our microarray experiment we could detect 292 up- and 244 down-regulated genes in *bli-1* seedlings (Figure 1; Tables 1, 2; TOP 25 up-regulated genes in Table 3). As BLI interacts with CLF and *bli-1 clf-28* double mutants revealed a synergistic genetic interaction (Schatlowski et al., 2010), we analyzed the overlap of differentially expressed genes in *bli-1* and *clf-28* mutants (Farrona et al., 2011). We found a significant overlap of differentially expressed genes between the two mutants (Figure 1) (Supplemental Data 2). Importantly, *CLF* is not differentially expressed in *bli-1* (Figure S2) and *BLI* is not differentially expressed in *clf-28* (Farrona et al., 2011). Among the commonly up-regulated genes in *bli-1* and *clf-28* are several H3K27me3 target genes (10 out of 18), e.g., the MADS-box transcription factor gene *SEPALLATA3* (*SEP3*). However, a large number of genes was only differentially expressed in one of either mutant. Because *CLF* function is masked by partial redundancy with *SWN*, we also compared the overlap of genes differentially expressed in *bli-1* and the strong *clf-28 swn-7* (*clf swn*) double mutant (Farrona et al., 2011), which is completely deficient in post-embryonic PRC2 function (Chanvivattana et al., 2004; Lafos et al., 2011). The overlap of genes up- regulated in *bli-1* and *clf swn* was significant (Figure 1C); among the 62 commonly differentially expressed genes 37 are targeted by H3K27me3. The overlap of down-regulated genes in *bli-1* and *clf swn* was also significant (Figure 1D); almost half of the down-regulated genes in *bli-1* were also down-regulated in *clf swn*, revealing a strong co-regulation of genes by BLI, CLF, and SWN. Among the 101 commonly down-regulated genes in *bli-1* and *clf swn*, 53 were H3K27me3 target genes. Our data hence reveal that a subset of genes targeted by CLF and/or SWN are co-regulated by BLI. Importantly, BLI likely also regulates genes in a PcG-independent manner.

**Table 1:** H3K27me3 target genes differentially expressed in *bli-1* seedlings.

	**PcG (H3K27me3) targets**	**Total no. genes**	**Percentage of H3K27me3 targets**	**Chi square test (*p*-value)**
Genome wide (Oh et al., 2008)	7,832^*^	27,235^**^	28.76	
*bli-1* up+down	208	536	38.81	0.0003
*bli-1* up	109	292	37.33	0.0241
*bli-1* down	98	244	40.16	0.0064

* *Genome wide H3K27me3 target genes refer to data from Oh et al. (2008)*.

** *Total number of protein coding genes according to TAIR8 genome release. Statistical significance was tested by Chi square test with Yates correction; a p-value equal to or below 0.05 was considered as statistically significant*.

**Table 2:** H3K4me3 target genes differentially expressed in *bli-1* seedlings.

	**H3K4me3 targets**	**Total no. genes**	**Percentage of H3K4me3 targets**	**Chi-square test (*p*-value)**
Genome wide (Roudier et al., 2011)	17,836	27,235^*^	65.49	
*bli-1* up+down	313	536	58.40	0.1182
*bli-1* up	172	292	58.90	0.2938
*bli-1* down	141	244	57.79	0.2600

* *Genome wide H3K4me3 target genes refer to data from Roudier et al. (2011)*.

**Total number of protein coding genes according to TAIR8 genome release. Statistical significance was tested by Chi square test with Yates correction; a p-value equal to or below 0.05 was considered as statistically significant.

**Table 3:** Top 25 up-regulated genes in bli-1 12 day old seedlings.

**#**	**Symbol**	**Description**	**Fold change**	**H3K27me3 target**
1	AT1G18830	Transducin/WD40 repeat-like superfamily protein; Secretory 31A (SEC31A)	124.35	Yes^**^
2	AT4G21730	Pseudogene of N-ethylmaleimide sensitive factor (NSF)	48.15	No
3	AT5G64060	NAC domain containing protein 103 (NAC103)	23.48	No
4	AT5G55270	Protein of unknown function (DUF295)	18.52	Yes
5	AT1G09080	Heat shock protein 70 (Hsp 70) family protein, Binding protein 3 (BIP3)	17.31	Yes^**^
6	AT1G17960	Threonyl-tRNA synthetase	14.13	Yes^*^
7	AT3G08970	DNAJ heat shock N-terminal domain-containing protein, (ERDJ3A)	12.39	No
8	AT2G29350	Senescence-associated gene 13 (SAG13)	10.90	Yes
9	AT5G53230	Protein of unknown function (DUF295)	10.73	Yes
10	AT5G53240	Protein of unknown function (DUF295)	9.96	Yes
11	AT1G09180	Secretion-associated RAS super family 1 (SARA1)	8.44	No
12	AT3G57260	beta-1,3-glucanase 2, PATHOGENESIS-RELATED PROTEIN 2, (PR2)	8.00	Yes
13	AT2G38240	2-oxoglutarate (2OG) and Fe(II)-dependent oxygenase superfamily protein	7.83	Yes
14	AT3G17050	Transposable element gene	7.65	Yes
15	AT3G55700	UDP-Glycosyltransferase superfamily protein	7.17	Yes^*^
16	AT5G64510	Tunicamycin-induced 1 (TIN1)	7.04	No
17	AT1G21528	Unknown protein	6.87	No
18	AT1G27020	Unknown protein	6.64	Yes
19	AT3G28899	Unknown protein	6.43	No
20	AT5G41761	Unknown protein	6.26	Yes
21	AT5G26270	Unknown protein	6.14	No
22	AT1G42990	basic region/leucine zipper motif 60 (bZIP60)	5.77	No
23	AT3G53232	ROTUNDIFOLIA like 1 (RTF1)	5.73	Yes
24	AT1G56060	Unknown protein	5.62	No
25	AT1G72280	Endoplasmic reticulum oxidoreductins 1 (ERO1)	5.37	No

Yes/no in the far-right column indicates if gene is a H3K27me3 target or not; Asterisks indicate genes that were tested for H3K27me3

(*) and H3K4me3

(**) *coverage in ChIP experiments*.

### *bli-1* mutants show a differential expression of H3K27me3 target genes but no loss of H3k27me3

**Figure 2:**
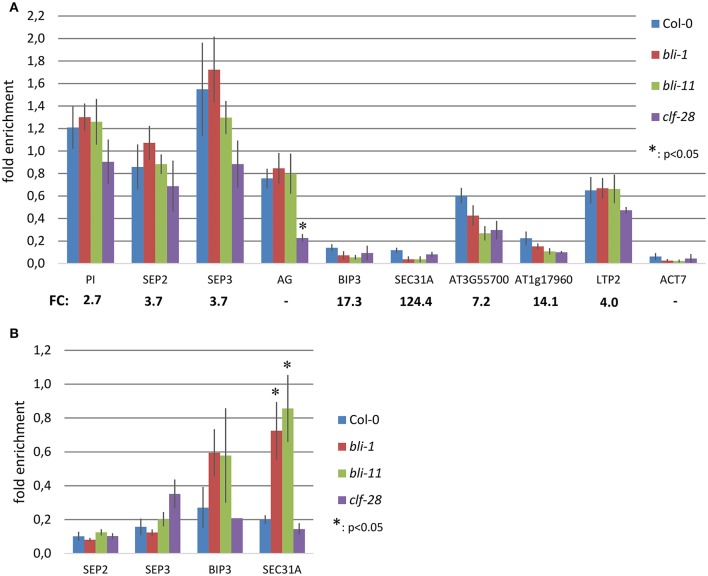
Chromatin immunoprecipitation (ChIP) in *bli* and *clf* mutants. **(A)** H3K27me3 levels at Polycomb target genes in 14 day old seedlings. Chromatin was precipitated using H3K27me3 antibodies and was amplified by quantitative PCR using oligonucleotides binding inside the gene body. H3K27me3 levels at each locus were normalized to the *FUS3* locus. FC: fold-change in expression level in *bli-1* compared to the wild type. **(B)** H3K4me3 levels at Polycomb target genes in 14 day old seedlings. Chromatin was precipitated using H3K4me3 antibodies and was amplified by quantitative PCR using oligonucleotides binding near the transcriptional start site. H3K4me3 levels at each locus were normalized to the *ACT7* locus. All ChIP experiments were performed twice with 2 biological and 3 technical replicates, respectively, and showed similar results. Error bars indicate ±SE of 2 independent experiments. Test for statistical significance by Student's *t*-test; a *p*-value below 0.05 was considered as statistically significant.

To further understand the role of BLI in PcG-mediated gene regulation, we compared the *bli-1* differentially expressed genes to PcG (H3K27me3) target genes. Indeed, we identified a significant number of PcG target genes differentially expressed in *bli-1* seedlings (Table 1, Supplemental Data 3), but no differential expression of PRC2 members (Supplemental Data 1). To further address the role of *BLI* in PcG mediated gene repression and reveal possible changes in H3K27me3 levels at differentially expressed genes, we performed Chromatin immunoprecipitation (ChIP) (Figure 2A). For ChIP experiments we used *bli-1, clf-28*, and the wildtype Col-0. Furthermore, we used the novel *bli-11* mutant, which strongly resembles *bli-1*, as an internal control to exclude possible T-DNA-dependent effects on *bli-1* chromatin modifications (for characterization of *bli-11* see Figure S3). We determined H3K27me3 levels at MADS-box transcription factor genes *PI* (*PISTILLATA*), *SEP2* (*SEPALLATA2*), and *SEP3*, which are well-known Pc-G target genes and are up-regulated in *bli-1*. Moreover, we determined H3K27me3 levels at several highly up-regulated Pc-G target genes in *bli-1*: *BIP3* (*BINDING PROTEIN3*), *SEC31A* (*SECRETORY31A*), At3g55700, At1g17960, and *LTP2* (*LIPID TRANSFER PROTEIN2*; Table 3). *AG* (*AGAMOUS*) is one of the main target genes of CLF and carries reduced H3K27me3 levels in *clf* mutants leading to ectopic expression (Goodrich et al., 1997; Schubert et al., 2006). In our ChIP experiments, both *bli* mutants did not show significant changes in H3K27me3 levels at all analyzed loci. *clf-28* showed significantly reduced H3K27me3 levels at *AG*, as expected, but not at other loci. In summary, we could not detect reduced levels of H3K27me3 at the tested loci in *bli-1* and *bli-11* mutants, despite a strong de-repression of these genes in *bli-1*, suggesting that differential expression of these genes is independent or downstream of H3K27me3. However, it is possible that changes in chromatin modifications at the tested loci are only occurring in specific tissues, which we would not detect in our analysis using whole seedlings.

The action of PcG proteins is counteracted by Trithorax group (TrxG) proteins, which set the activating H3K4me3 mark. H3K4me3 targets a much higher number of genes (~50% of the Arabidopsis genome) than H3K27me3 (Oh et al., 2008; Zhang et al., 2009; Bouyer et al., 2011; Roudier et al., 2011). To test if the increased expression of PcG-target genes in *bli-1* mutants is due to increased activity of TrxG proteins—hence elevated levels of H3K4me3 at those genes—we performed a ChIP experiment using antibodies directed against H3K4me3 (Figure 2B). For H3K4me3 ChIP experiments we analyzed two PcG target genes carrying a high level of H3K27me3 (*SEP2, SEP3*) and two carrying a low H3K27me3 level (*BIP3, SEC31A*). Only the highest expressed gene in *bli-1, SEC31A* (Table 3), showed significantly increased H3K4me3 levels (Figure 2B), suggesting that elevated expression of PcG target genes in *bli* mutants is not necessarily related to an increased H3K4me3 accumulation. Generally, genes targeted by H3K4me3 were not enriched among differentially expressed genes in *bli-1* compared to the number of H3K4me3 target genes in the genome (Table 2).

### *bli* plants show additional expression domains of *CLV3* and *CYCB1;1*

In the severe *clf swn* double mutant H3K27me3 is completely lost (Lafos et al., 2011). Cell fate decisions in this mutant cannot be maintained throughout development, leading to a loss of cell identity and the formation of callus-like tissue (Chanvivattana et al., 2004). Presence of blister-like structures in *bli-1* (Schatlowski et al., 2010) and *bli-11* mutants (Figures S3F,G) indicate a loss of cell identity in *bli* mutants. The blister-like structures may have meristematic activity or are actively dividing cells in an otherwise differentiated tissue. However, transcriptional profiling of *bli-1* did not reveal changes in the expression of the stem cell marker *CLV3* (*CLAVATA 3*) and the cell division marker *CYCB1;1* (*CYCLIN-DEPENDENT PROTEIN KINASE B1;1*), possibly because differential expression of a gene in a small population of cells might not be detected when whole seedlings are used for transcriptional profiling. To test if this could be the case for *bli* mutants, we analyzed the expression pattern of a *CLV3:GUS* and a *CYCB1;1:GUS* reporter gene in the *bli-11* mutant. We used *bli-11* for GUS reporter assays since *bli-1* shows ectopic expression of *LAT52:GUS* present on the SAIL T-DNA integrated in the *BLI* locus (Schatlowski et al., 2010).

**Figure 3:**
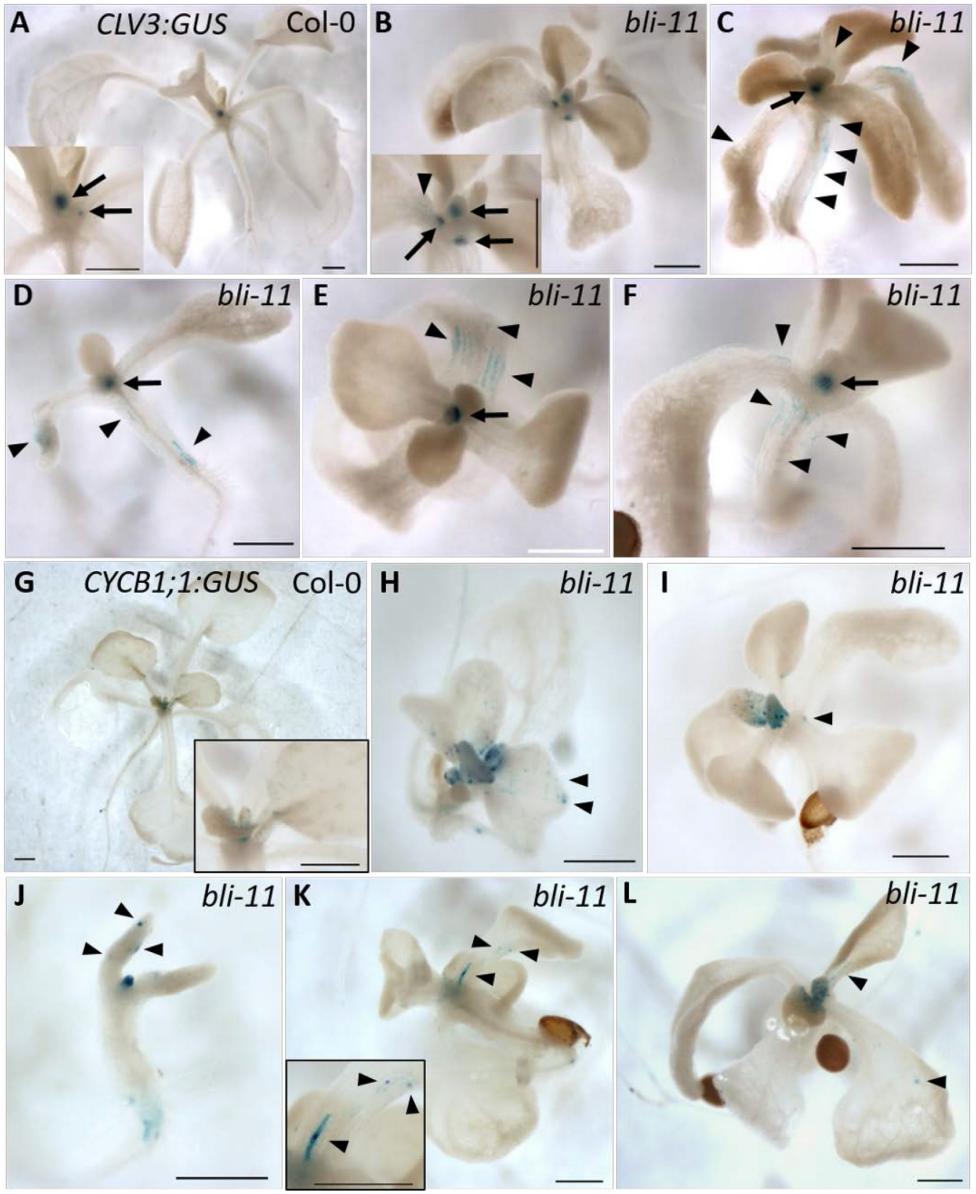
Expression of *CLV3:GUS* and *CYCB1;1:GUS* in *bli-11* mutants. **(A)** Col-0 seedlings showed SAM-specific *CLV3:GUS* expression. **(B–F)** 43% of *bli-11* mutants showed an ectopic expression of *CLV3:GUS*, revealing a loss of cell identity. Arrows point to meristems (SAM and axillary) and arrowheads mark ectopic *CLV3:GUS* expression. **(G)** Col-0 expressing *CYCB1;1:GUS*. **(H–L)** 32% *bli-11* seedlings showed ectopic expression of *CYCB1;1:GUS*. Arrowheads mark ectopic *CYCB1;1:GUS* expression. Scale bars are 500 μm.

*CLV3:GUS* showed ectopic expression in 43% (32 out of 74 seedlings) of *bli-11* seedlings, mainly in hypocotyls and cotyledons (Figures 3B–F). *CYCB1;1:GUS* was also ectopically expressed in *bli-11* (32%, 8 of 25 seedlings) (Figures 3H–L), particularly in differentiated leaves in which *CYCB1;1* expression has seized in wild type plants. Expression of both reporters was confined to a limited number of cells, which may reflect blister-like structures or de-differentiating cells. Overall, ectopic expression of the stem cell marker *CLV3* and the cell division marker *CYCB1;1* in *bli-11* mutants indicate that *BLI* acts in maintaining cell identity and in suppression of improper or ectopic cell-divisions.

### Stress-responsive genes are strongly up-regulated in *bli-1* mutants

In order to functionally characterize the differentially expressed genes in *bli-1*, we performed a GO-term analysis. We found enrichment of several GO-terms for stress-responses among the up-regulated genes in *bli-1*; a subset of these GO-terms is displayed in Table 4 (for full list of GO terms see Supplemental Data 4). The most significantly enriched GO-terms for a specific form of stress were “response to endoplasmic reticulum stress” (GO-ID: 0034976) and “endoplasmic reticulum unfolded protein response” (GO-ID: 0030968). This might indicate that BLI plays a role in the regulation of the ER-stress response/UPR (unfolded protein response). The GO-term “response to heat” (GO-ID: 0009408) was also enriched among up-regulated genes in *bli-1*.

**Table 4:** Selected GO-IDs enriched in up- and down-regulated genes in *bli-1*.

**GO-ID**	**Term-name**	***p*-value**
**UP-REGULATED IN** ***BLI-1***		
GO:0006950	Response to stress	9.75E-11
GO:0034976	Response to endoplasmic reticulum stress	4.09E-11
GO:0030968	Endoplasmic reticulum unfolded protein response	0.000484
GO:0009408	Response to heat	0.005793
GO:0009414	Response to water deprivation	0.012527
GO:0009737	Response to abscisic acid stimulus	0.018293
GO:0009651	Response to salt stress	0.040284
**DOWN-REGULATED IN** ***BLI-1***		
GO:0010374	Stomatal complex development	6.75E-06
GO:0048367	Shoot development	0.000113
GO:0048366	Leaf development	0.000372
GO:0008544	Epidermis development	0.000588
GO:0042335	Cuticle development	0.003212
GO:0009409	Response to cold	0.022874
GO:0009611	Response to wounding	0.029012

*Statistical significance was determined using the hypergeometric test with Benjamini-Hochberg correction; a p-value below 0.05 was considered as statistically significant. A GO Slim analysis can be found in Figure S1*.

The “response to abscisic acid stimulus” (GO-ID: 0009737) was also significantly enriched among *bli-1* up-regulated genes. The phytohormone abscisic acid (ABA) promotes seed dormancy and desiccation tolerance and regulates embryo and seed development. In adult plants ABA regulates general growth and reproduction and is induced by abiotic stresses, such as drought, high salinity, and cold, and hence considered a “stress hormone” (reviewed in Tuteja, 2007). In *bli-1* seedlings we found a significant differential expression of ABA-responsive genes (Zeller et al., 2009; Table 5, full list in Supplemental Data 5). Additionally, a significant number ABA-responsive genes is also targeted by H3K27me3. This suggests an important function for BLI in regulating ABA-responsive PcG target genes (Table 5). Interestingly, among the 18 commonly up-regulated genes in *bli-1* and *clf-28* mutants, 7 were regulated by ABA. Among differentially expressed ABA-responsive genes we did not detect key regulators of ABA biosynthesis or catabolism, or ABA perception and transport. This indicates that down-stream processes of ABA signaling, possibly genes transcriptionally regulated by ABA signaling, are affected in *bli-1*. As ABA regulates responses to drought stress and high salinity, it is consistent that the GO-terms “response to water deprivation” (GO-ID: 0009414) and “response to salt stress” (GO-ID: 0009651) were also significantly enriched among *bli-1* up-regulated genes. A detailed analysis of the genes belonging to the GO-term “response to water deprivation” revealed that most of these genes are directly regulated by ABA and targeted by H3K27me3. Taken together, up-regulation of genes in *bli* mutants which are regulated in “response to abscisic acid stimulus,” “response to water deprivation,” and “response to salt stress” indicates a role of *BLI* in ABA-dependent gene regulation.

**Table 5:** Differential expression of ABA-responsive and SAR genes in *bli-1* mutants.

	**ABA-responsive genes**	**Total no. genes**	**percentage of ABA-responsive genes**	**Chi square test (*p*-value)**
Genome wide (Zeller et al., 2009)	2,197	27,235^*^	8.07	
*bli-1* up+down	98	536	18.28	<0.0001
*bli-1* up	55	292	18.84	<0.0001
*bli-1* down	43	244	17.62	<0.0001
*bli-1* H3K27me3 target genes	47	208	22.60	<0.0001
	**SAR (up)**		**Percentage of SAR genes**	
Genome wide (Gruner et al., 2013)	547	27,235^*^	2.01	
*bli-1* up	56	292	19.18	<0.0001
*bli-1* down	2	244	0.82	0.2840

In bli-1 a significant number of ABA-responsive genes (Zeller et al., 2009) is differentially expressed. Also differentially expressed H3K27me3 target genes in bli-1 are enriched for ABA responsive genes. A significant number of genes up-regulated in bli-1 was also up-regulated by SAR (Gruner et al., 2013).

* *Total number of protein coding genes according to TAIR8 genome release. Statistical significance was determined using Chi square test with Yates correction; a p-value below 0.05 was considered as statistically significant*.

GO-term analysis of down-regulated genes in *bli-1* revealed strong enrichment of developmental processes, such as “stomatal complex development” (GO-ID: 0010374), “shoot development” (GO-ID: 0048367), and “leaf development” (GO-ID: 0048366) (Table 4). Our previous study indeed showed affected shoot and leaf development in *bli-1* (Schatlowski et al., 2010). Moreover, we previously showed that epidermis and cuticle development are affected in *bli-1*, resulting in gaps in the epidermis (Schatlowski et al., 2010). Consistent with this observation, we found the GO-terms “epidermis development” (GO-ID: 0008544), “cuticle development” (GO-ID: 0042335), and “response to wounding” (GO-ID: 0009611) among the down-regulated genes in *bli-1* (Table 4). A study by Purdy et al. (2010) showed that the induction of cold stress-responsive genes was impaired in *bli* mutants exposed to prolonged cold. Conclusively, the GO-term “response to cold” (GO-ID: 0009409) was enriched among down-regulated genes in *bli-1* revealing that, even under ambient temperatures, the expression of cold regulated genes is affected. Taken together, the GO term analysis of up- and down-regulated genes in *bli-1* strongly indicates a role for BLI in repression of stress-responsive genes which are to a large extent H3K27me3 targets and promotion of genes involved in developmental control.

**Figure 4:**
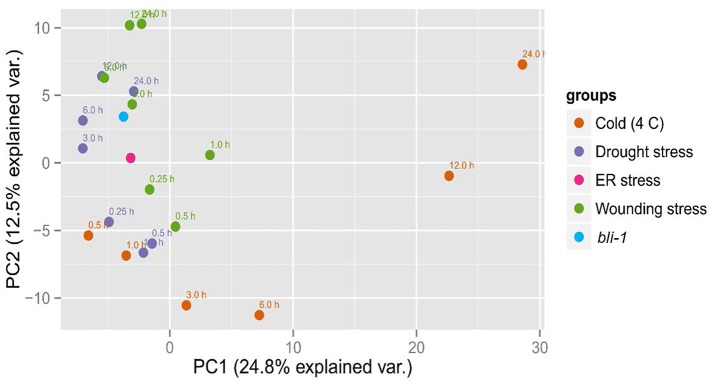
Principal Component Analysis (PCA) of *bli-1* mutants and several stress responses. Results from the *bli-1* microarray were compared to cold-, drought-, and wounding responses (Kilian et al., 2007) as well as to ER-stress (Nagashima et al., 2011). In the cases of stress treatment, expression was normalized against control, while the data on *bli-1* was normalized against wild type. We performed Principal Component Analysis on the log2-tranformed fold changes in gene expression (see Section Materials and Methods).

To confirm that BLI plays an important role in the regulation of stress responses we performed Principal Component analysis (PCA) on the expression patterns of *bli-1* and responses to cold, drought, wounding (Kilian et al., 2007) and ER-stress (Nagashima et al., 2011; Figure 4). *bli-1* clustered strongly with responses to prolonged drought and wounding and with ER-stress. PC1 separated *bli-1* from prolonged cold stress (>3 h) as well as short-term wounding responses. PC2 separated *bli-1* from short-term responses to cold, drought, and wounding. PC1 and PC2 could explain about 25 and 13% of the observed variance in the data, respectively, hence showing that those PCs were relevant for revealing differences between samples/treatments. The results of our PCA further indicate that BLI is an important regulator of genes involved in several stress responses.

Finally, we tested if BLI is also involved in biotic stress responses and compared differentially expressed genes in *bli-1* with genes up-regulated by systemic acquired resistance (SAR) (Gruner et al., 2013). We found that a high number of genes up-regulated by SAR were also up-regulated in *bli-1* (Table 5). Interestingly, out of the 56 genes commonly up-regulated in *bli-1* and by SAR, 23 are also up-regulated in response to ER-stress. This once again shows that stress responses in plants are interconnected, and that a regulator of one stress response can regulate several linked pathways or rather commonly regulated genes.

### *bli* mutants are hypersensitive to desiccation stress

**Figure 5:**
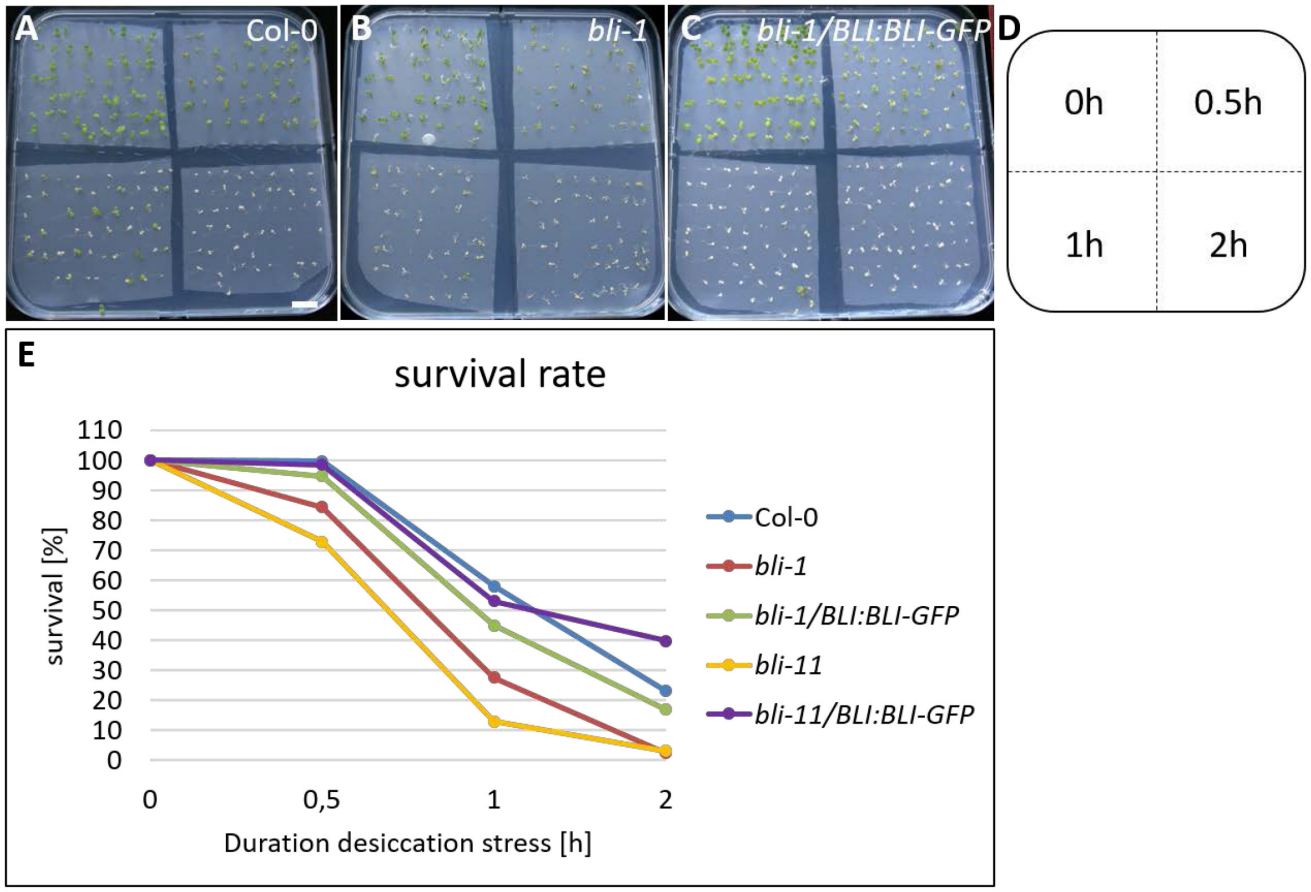
Desiccation stress treatment of *bli* mutants and complemented lines. Five day old seedlings underwent 0, 0.5, 1, and 2 h of desiccation stress **(A–D)** and were scored for survival 5 days after stress treatment **(E)**. Survival of all genotypes was strongly reduced with increasing duration of desiccation stress treatment. Scale bar is 1 cm.

The GO-term analysis and PCA strongly indicated that *BLI* plays an important role in regulation of stress responses. Because *bli-1* clustered strongly with long term drought stress responses in the PCA, we aimed to analyze the ability of *bli* mutants to cope with desiccation. For that purpose, we subjected two strong *bli* mutants, *bli-1* and *bli-11*, to different periods of desiccation stress (Table 6; for experimental setup see Figure S4). The stress treatment revealed that both *bli* mutants were hypersensitive to desiccation (Table 6 and Figure 5). Both complemented lines were able to rescue the desiccation-sensitive *bli* phenotype under the tested conditions, although the *bli-1/BLI:BLI-GFP* line showed a mild desiccation sensitivity after 0.5 and 1 h of desiccation stress, suggesting only partial complementation. These results show that loss of *BLI* reduces the ability of *bli* mutants to survive under desiccation stress conditions.

**Table 6:** Survival of *bli* mutants and complemented lines after different periods of desiccation stress.

**Exposure to drought (h)**	**0**	**0.5**			**1**			**2**		
**Genotype**	**Viable**	**Viable**	**Dead**	**Fishers exact test *p*-value**	**Viable**	**Dead**	**Fishers exact test *p*-value**	**Viable**	**Dead**	**Fishers exact test *p*-value**
Col-0	398	394	1		198	143		83	275	
*bli-1*	283	242	45	0.0001	73	192	0.0001	7	285	0.0001
*bli-1*/BLI:BLI-GFP	314	320	18	0.0001^*^	147	180	0.0001^*^	54	264	0.0001^*^
*bli-11*	231	126	47	0.0001	22	148	0.0001	5	150	0.0001
*bli-11*/BLI:BLI-GFP	349	340	5	0.0001^*^	170	150	0.0001^*^	137	207	0.0001^*^

Five day old seedlings underwent 0, 0.5, 1, and 2 h of desiccation stress (see Figure S4 for experimental setup) and were scored for survival 5 days after stress treatment. Four independent experiments with each two biological replicates were combined here. Ratios of all bli mutants were compared to the wild type.

* *Ratios of complemented lines were compared to the respective mutant, to test the complementation ability. Statistical significance was analyzed using fishers exact test; a p-value equal to or below 0.05 was considered as statistically significant*.

## Discussion

### Regulation of PCG target genes by BLI

**Figure 6:**
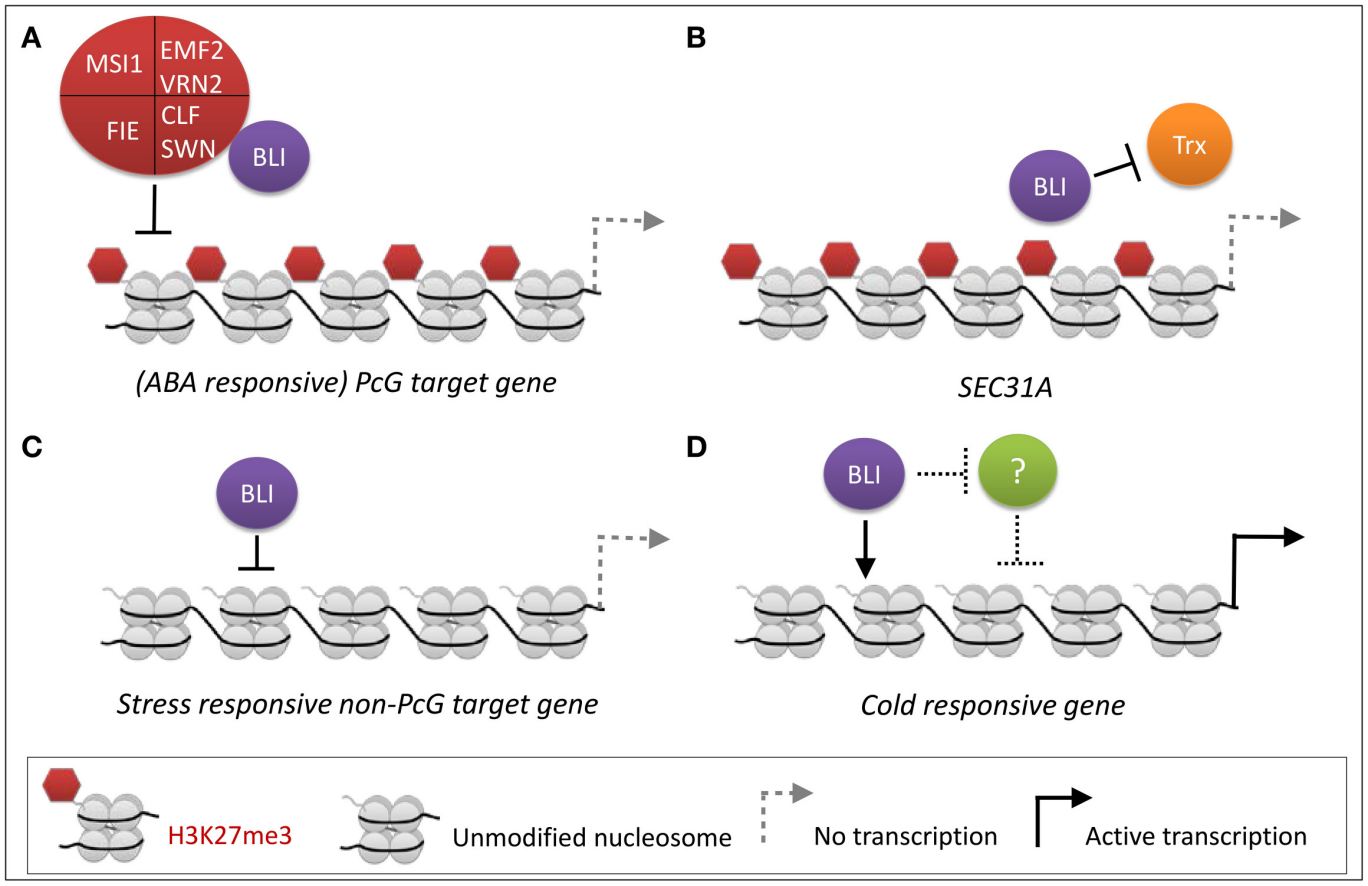
PcG-dependent and -independent regulation of stress-responsive gene expression by BLI. **(A)** PcG-dependent functions of BLI. BLI silences (ABA-responsive) PcG target genes, likely in parallel or downstream of PRC2. **(B)** BLI likely prevents activation of certain PcG target genes by TrxG proteins. **(C,D)** PcG-independent functions of BLI. **(C)** BLI represses stress-responsive non-PcG target genes under normal growth conditions. This could also be an indirect effect. **(D)** During cold stress BLI activates gene expression of cold-responsive genes, such as *COR15A* (Purdy et al., 2010), or represses an unknown repressor (indicated as “?”) of cold-responsive genes.

We previously identified BLI as an interactor of the PRC2 methyltransferase CLF (Schatlowski et al., 2010). To further dissect the role of BLI in PcG-related and -unrelated functions, we analyzed the transcriptome of *bli-1* seedlings and found a significant overlap of genes regulated by BLI and CLF. However, a high number of genes was not co-regulated by BLI and CLF, possibly because BLI has PcG-independent functions, or the function of CLF is masked by its partial redundancy with SWN. To account for the latter, the overlap of genes regulated by BLI and CLF/SWN was analyzed, revealing a stronger co-regulation of genes by BLI and CLF/SWN as for CLF alone. Nevertheless, the overlap in co-regulated genes was relatively small, possibly due to the fact that the gene expression analyses in *clf*, *clf swn*, and *bli* was not performed in identical conditions, in a tissue-specific manner and/or did not take all Arabidopsis transcripts into account due to the array-based transcriptomic analyses. Our analyses still indicate, however, that BLI plays an important role in regulating a subset of genes targeted by PRC2 containing CLF or SWN. Importantly, transcriptional profiling of *bli-1* revealed a significant differential expression of PcG target genes, but ChIP experiments revealed neither reduction nor loss of H3K27me3 levels at these loci (Figure 2A) as we had previously revealed for additional loci (Schatlowski et al., 2010). Silencing of PcG target genes is not only dependent on PRC2 but also on PRC1, and other PcG proteins. Levels of H3K27me3 are affected in all analyzed PRC1 mutants but not at all PcG target genes (Calonje et al., 2008; Derkacheva et al., 2013; Yang et al., 2013; Wang et al., 2016). The PRC1 protein EMF1 is an interactor of MSI1 (Calonje et al., 2008) and like PRC2 mutants, *emf1* mutants show reduced H3K27me3 levels, but only at a subset of PRC2 target genes such as *AG* but not at *FUS3* (Calonje et al., 2008; Kim et al., 2012; Yang et al., 2013). The fact that levels of H3K27me3 at all analyzed loci are neither decreased nor increased in *bli-1* suggests that BLI is likely not involved in PRC2 recruitment or in H3K27me3 maintenance but it is also possible that H3K27me3 occupancy is only affected in a cell-type specific manner which we would not have detected in our analyses. In addition, genome-wide analyses of H3K27me3 occupancy may identify genes which show altered H3K27me3 in *bli* mutants. Differential expression of PcG target genes in *bli-1* however indicates that BLI most likely regulates PcG target gene expression downstream of, or in parallel to, PRC2. Additionally, BLI likely also regulates gene expression independently of the PcG system (Figure 6C). To dissect direct and indirect effects of BLI, ChIP-seq analyses of BLI will be required in the future.

As the action of PcG proteins is counteracted by Trithorax group proteins, we also tested H3K4me3 coverage of several up-regulated PcG target genes in *bli-1* mutants. Our analysis indicated that BLI is at least partially responsible for prevention of gain or increase of H3K4me3 at certain PcG target genes, such as at the *SEC31A* locus (Figure 2B, Figure 6B). A recent study showed that during drought stress treatment levels of H3K27me3 remained constant at PcG target genes, while H3K4me3 levels increased resulting in active transcription (Liu et al., 2014). Therefore, BLI might restrict binding of TrxG proteins to certain PcG target genes to prevent switches from repressive to active chromatin states during normal growth or under stress conditions (Figure 6B). As BLI contains coiled-coil domains it may be involved in stabilizing the chromatin in non-stress conditions to prevent precocious expression of stress-inducible genes. Future analysis of direct target genes and interaction partners of BLI will reveal if BLI directly interacts with PRC1 or TrxG proteins to stably silence genes or to restrict their activation, respectively.

### BLI regulates specific developmental pathways

The strong *clf swn* or *vrn2 emf2* double mutants cannot sustain cell fate decisions during development, and develop into a callus-like cell mass early during seedling development (Chanvivattana et al., 2004; Schubert et al., 2005). Blister-like structures on several organs of *bli* mutants indicate a loss of cell identity. Moreover, *bli* mutants show enhanced endoreduplication and fewer cells, indicating a role for BLI in cell division regulation or cell cycle regulation. The stem cell marker *CLV3* and cell division marker *CYCB1;1* showed small domains of ectopic expression in *bli* mutants (Figure 3). *CLV3* is a PcG target gene encoding a precursor of a small secreted peptide which regulates SAM size (Fletcher et al., 1999; Brand et al., 2000). Thus, regulation of *CLV3* expression is likely a PcG-dependent function of BLI. *CYCB1;1* is highly expressed in the G2/M phase of the cell cycle and is not a PcG target. Repression of *CYCB1;1* might hence be a PcG-independent function of BLI. Taken together, ectopic expression of *CLV3* and *CYCB1;1* in *bli* mutants suggests that BLI is a negative regulator of differentiation by preventing ectopic meristematic activity and endoreduplication without cell division.

### Role of BLI in abiotic stress responses

Transcriptional profiling of *bli-1* mutants revealed a strong enrichment of stress-responsive genes among the up-regulated genes, suggesting that BLI prevents precocious expression of stress-responsive genes. We found that genes involved in response to ER-stress, drought, high salt, heat, and genes up-regulated by SAR were up-regulated in *bli*, whereas responses to cold and wounding were enriched among down-regulated genes (Tables 4, 5). A principal component analysis (PCA) showed that *bli-1* expression profiles clustered with responses to drought, ER-stress, wounding, and, to a lesser extent, cold (Figure 4). Stress responses are cost-intensive, require extensive protein production in order to compensate for the stress, and consume important resources of a plant, which are required for growth and reproduction. Under ambient conditions it is important for a plant to prevent cost-intensive stress responses. To achieve this, stress responses are only induced in response to stress and are suppressed under non-stress conditions, a function that is apparently partially dependent on BLI.

The responses to drought and heat are connected: the transcription factor DREB2A was shown to have dual function in responses to drought and heat (Sakuma et al., 2006). Additionally, the drought-stress-responsive transcription factor NAC019, which is one of the up-regulated PcG target genes in *bli-1*, was recently reported to be heat-stress-responsive (Sullivan et al., 2014). The same study also discovered that *BLI* expression is highly increased in response to heat-stress (Sullivan et al., 2014). Up-regulation of genes induced by drought and heat in *bli* mutants indicate that BLI negatively regulates these responses. BLI might repress cost-intensive responses to these stressors during non-stress conditions which might explain why *bli* mutants are hypersensitive to desiccation stress: if the mutant already suffers from cost-intensive stress responses, additional stress treatment would lead to an inability to further respond to this stress, ultimately killing the plant.

Responses to cold, drought, and high salt are mediated by abscisic acid (ABA)-dependent but also ABA-independent pathways. In *bli-1* the GO-term “response to abscisic acid stimulus” was enriched among up-regulated genes, and consistently a significant number of ABA-responsive genes differentially expressed in *bli-1* (Table 5). Additionally, a significant number of differentially expressed ABA-responsive genes is targeted by H3K27me3 (Table 5), indicating that BLI might be involved in the regulation of ABA-responsive PcG target genes (Figure 6A).

The role of PcG proteins in stress responses is only emerging (reviewed in Kleinmanns and Schubert, 2014). PRC2 and PRC1 proteins were shown to be involved in the regulation of stress-responsive genes or regulators of stress responses. For example, the PRC1 RING-finger proteins AtBMI1a and AtRING1b, also known as DREB2A-INTERACTING PROTEIN 2 (DRIP2) and DRIP1, respectively, are important negative regulators of drought-responsive gene expression by targeting DREB2A to 26S proteasome-mediated proteolysis (Qin et al., 2008). However, the role of AtBMI1a and AtRING1b in PcG-dependent silencing of drought-stress-responsive genes has not been resolved. PRC1 proteins EMF1 and EMF2 repress several categories of stress-induced genes such as cold-stress induced *COR15A* (Kim et al., 2010). Under non-stress conditions EMF1 directly binds to genes involved in biotic and abiotic stress, and these binding sites largely overlap with H3K27me3 sites (Kim et al., 2012). MSI1 was shown to be a negative regulator of drought stress responses; the *msi1* co-suppressed mutant *msi1-cs* was reported to be more resistant to drought stress (Alexandre et al., 2009). Recently, a study revealed that MSI1 functions in a histone deacetylase complex to fine-tune ABA signaling and that loss of MSI1 led to an increased tolerance to salt stress (Mehdi et al., 2016). In the study by Mehdi et al. (2016) it was shown that MSI1 binds to chromatin of ABA receptor genes *PYL4, PYL5, PYL6*, and that loss of MSI1 decreased levels of H3K9 acetylation at those loci. The level of H3K27me3 were not analyzed in the studies by Alexandre et al. (2009) and Mehdi et al. (2016), therefore it remains unclear if the PcG function of MSI1 plays a role in the regulation of stress-responsive genes. In contrast to *msi1-cs, clf* mutants showed a reduced resistance to drought (Liu et al., 2014). Interestingly, ABA levels were reduced during normal growth and during stress treatment in *clf* mutants (Liu et al., 2014). This indicates that during drought stress ABA-responsive genes might not be properly induced in the *clf* background, hence leading to reduced drought stress tolerance. Since genes involved in ABA biosynthesis or catabolism, or ABA reception or transport were not differentially expressed in *bli-1*, the reduced desiccation tolerance is likely due to a different mechanism than in *clf*. CLF and BLI are both necessary to cope with drought/desiccation stress, and probably cooperatively regulate certain ABA-responsive PcG target genes (Figure 6A). However, BLI likely also regulates ABA-responsive genes independent of the PcG system (Figure 6D).

In summary, our transcriptional profiling revealed that BLI regulates a subset of PcG target genes. Since H3K27me3 levels were not altered in *bli-1* mutants, BLI likely acts downstream of, or in parallel to PRC2 in gene silencing. Moreover, we identified BLI as a regulator of several stress responses, which is at least partially a PcG-dependent function. Therefore, BLI may be a key protein in connecting chromatin-mediated integration of stress responses, a process that is not well understood in plants. Analysis of BLI target genes and interaction partners under ambient and stress conditions will reveal which role BLI plays in PcG-dependent and -independent regulation of stress-responsive genes.

## Author contributions

JK, NS, and DS designed research. NS prepared RNA samples and performed the microarray experiment. DH processed the microarray data and performed the PCA. JK evaluated the microarray and performed all other experiments. JK and DS wrote the paper.

### Conflict of interest statement

The authors declare that the research was conducted in the absence of any commercial or financial relationships that could be construed as a potential conflict of interest.

## Footnotes

^1^with contributions from James MacDonald Jeff Gentry. gcrma: Background Adjustment Using Sequence Information. In R package version 2380.
