# Supplementary material for: BLISTER Regulates Polycomb-Target Genes, Represses Stress-Regulated Genes and Promotes Stress Responses in Arabidopsis thaliana
Source: Front Plant Sci. 2017 Sep 11;8:1530. doi: 10.3389/fpls.2017.01530 (PMC5601981; doi:10.3389/fpls.2017.01530)
Supplement: Supplementary file 1 [file SupplementaryInformation.PDF]

“BLISTER regulates Polycomb-target genes, represses stress-regulated genes and promotes stress responses in *Arabidopsis thaliana*”

Julia Anna Kleinmanns, Nicole Schatlowski, David Heckmann and Daniel Schubert

Supplemental Information

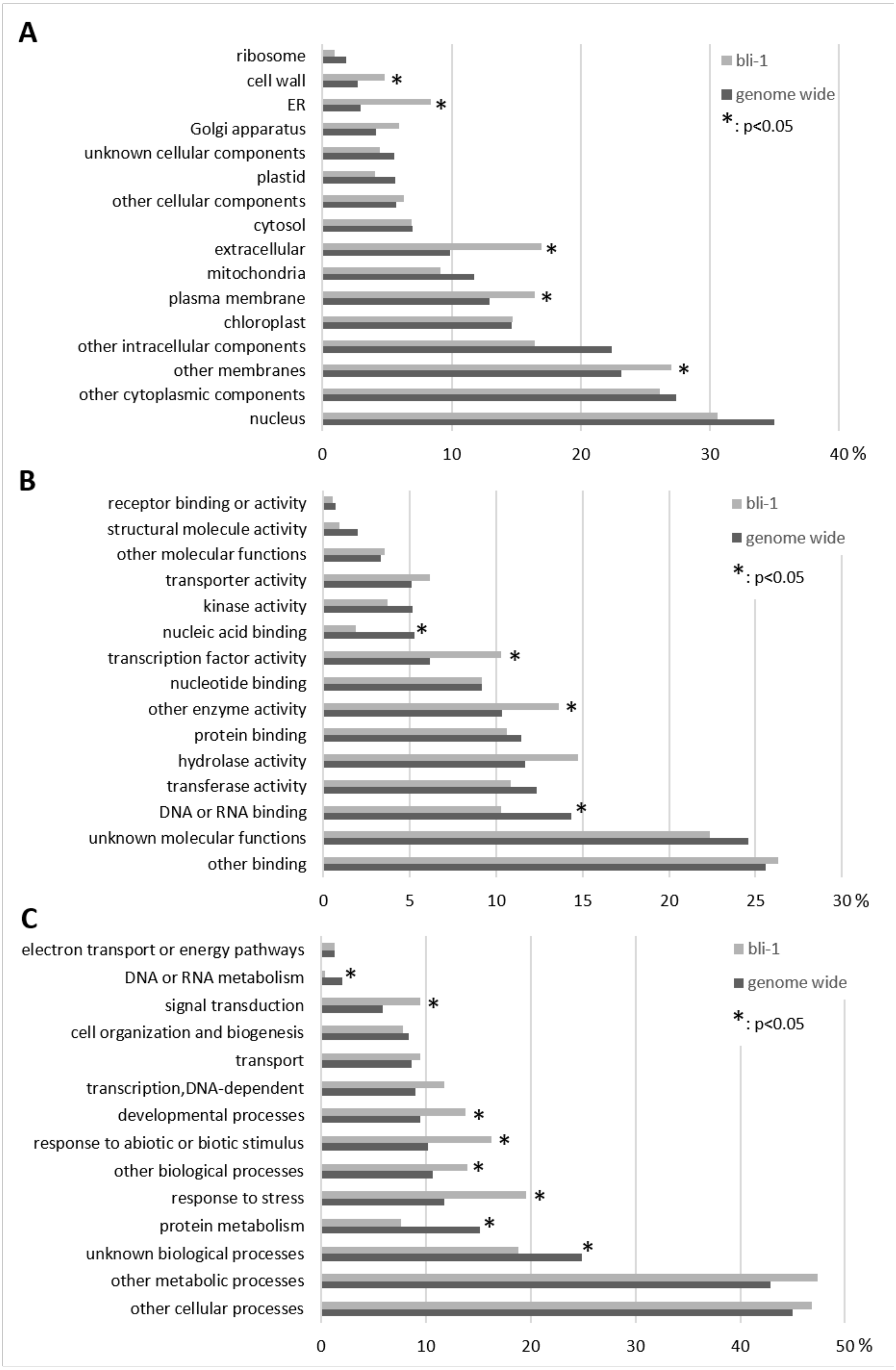

Figure S1: GO Slim analysis for all up- and down-regulated genes in *bli-1* seedlings vs. genome wide.

A) GO Cellular Component. B) GO Molecular Function. C) GO Biological process. Asterisks indicate significant changes. Test for statistical significance by Chi square test with Yates correction; a p-value below 0.05 was considered as statistically significant.

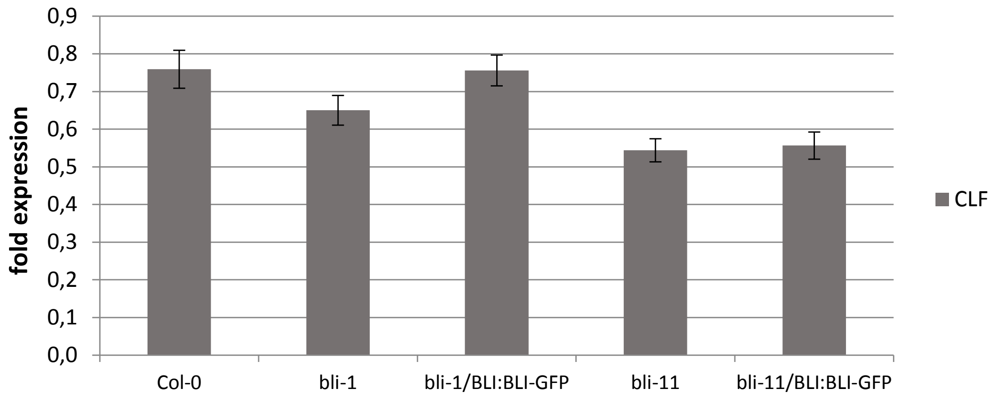

Figure S2: Expression of *CLF* in *bli* mutants and complemented lines.

CLF transcription is not changed in *bli* mutants or complemented lines compared to the wild type. Statistical significance was analysed using Student´s t-test; a p-value below 0.05 was considered as statistically significant.

Characterization of the novel *bli-11* mutant

In this study, we characterized a novel *bli* allele, *bli-11* (GABI-Kat_663H12). *bli-11* is the only available *BLI* allele comprising a T-DNA insertion in an exon (exon no. 7) (Figure S3 J). Only *bli-1* and *bli-11* contain a T-DNA insertion in the highly conserved SMC-like domain, which is the domain important for interaction with the PRC2 member CURLY LEAF (CLF) (Schatlowski et al., 2010). Analysis of *BLI* transcript level in *bli* mutants, revealed a reduced transcription in *bli-11* and no transcript in *bli-1* (Figure S3 H, J). Importantly, we were unable to detect a full length transcript containing the SMC-like domain in *bli-11* and *bli-1* (Figure S3 I, J), rendering *bli-11* a null or severe loss-of-function mutant. The *bli-11* mutant phenotypically resembles the *bli-1* mutant, showing a strong pleiotropic phenotype and blister-like structures on several organs (Figure S3 F, G). Introduction of a genomic copy of BLI fused to GFP (*BLI:BLI-GFP*) could rescue the *bli-11* phenotype, showing that loss of *BLI* function was causing the observed *bli-1*-like phenotype of *bli-11* (Figure S3 K). Because of the strong similarity of the severe loss-of-function mutant *bli-1* and *bli-11*, we included *bli-11* in our experiments.

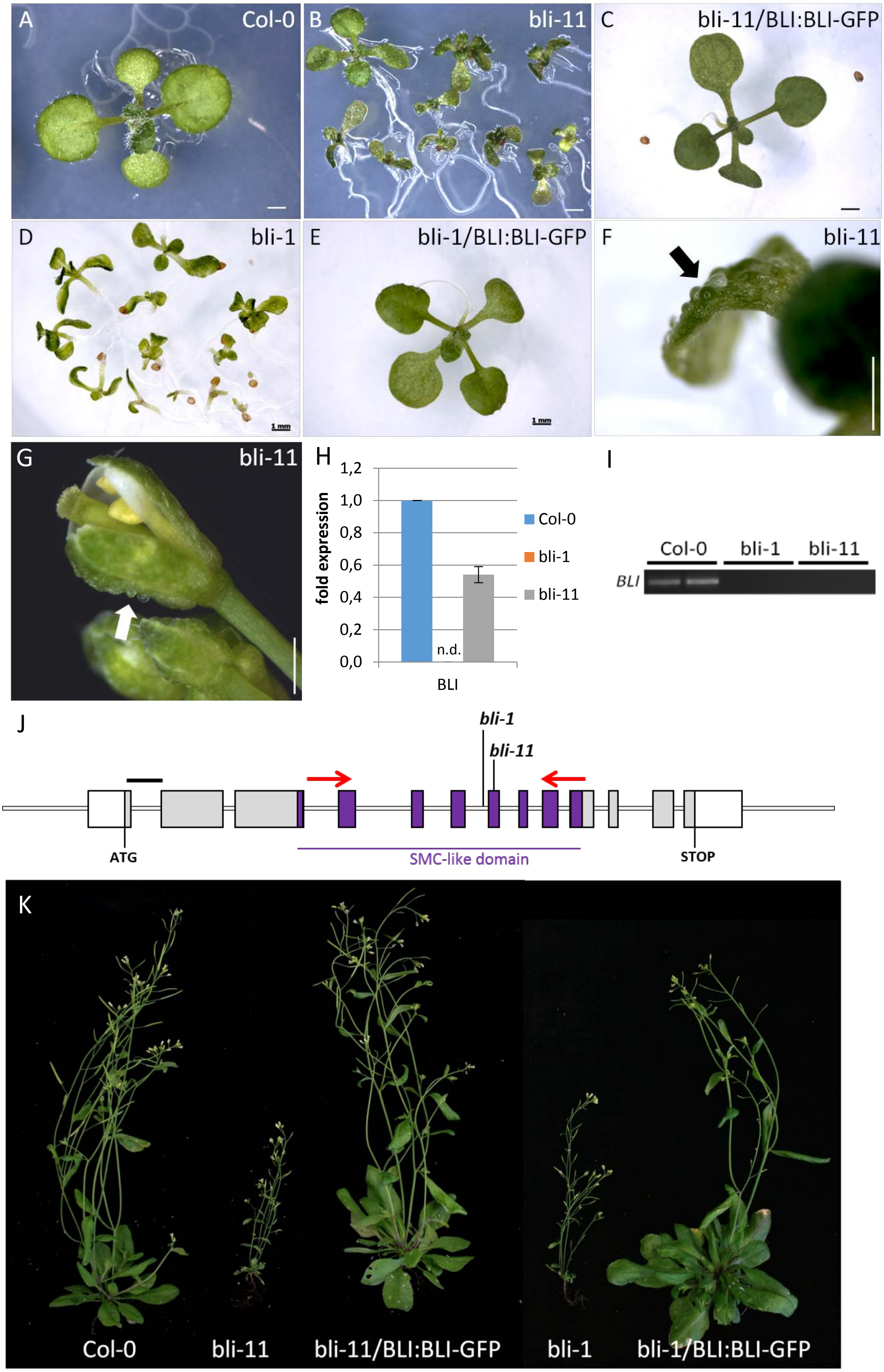

Figure S3: Characterization of the novel *bli-11* mutant.

A-E) The *bli-11* mutant strongly resembles *bli-1*. Both mutants could be complemented by introduction of full-length genomic BLI fused to GFP (BLI:BLI-GFP) (K). F-G) *bli-11* mutants exhibit blister-like structures on cotyledons and flowers, a phenotype also observed in *bli-1*. H) The expression analysis revealed reduced *BLI* transcription in *bli-11* and no transcription in *bli-1* (n.d.: not detected; see horizontal black line in J for amplification site). Expression was normalized to *ACT2*, 2 biological replicates with 3 technical replicates each, ± SE of biol. replicates. I) No full-length transcript (region between red arrows in J) could not be detected in either *bli-11* or *bli-1*, indicating a non-functional *BLI* gene in both mutants (two biological replicates for each genotype are shown). J) *BLI* locus. Light grey and purple boxes indicate exons. Purple coloured boxes indicate exons coding for the conserved SMC-like domain, important for interaction with CLF. Horizontal black line indicates fragment amplified in H); red arrows show region amplified to test for full-length transcript in I). K) Adult *bli* mutants and the respective complemented lines. A *BLI:BLI-GFP* transgene could complement the *bli* phenotype.

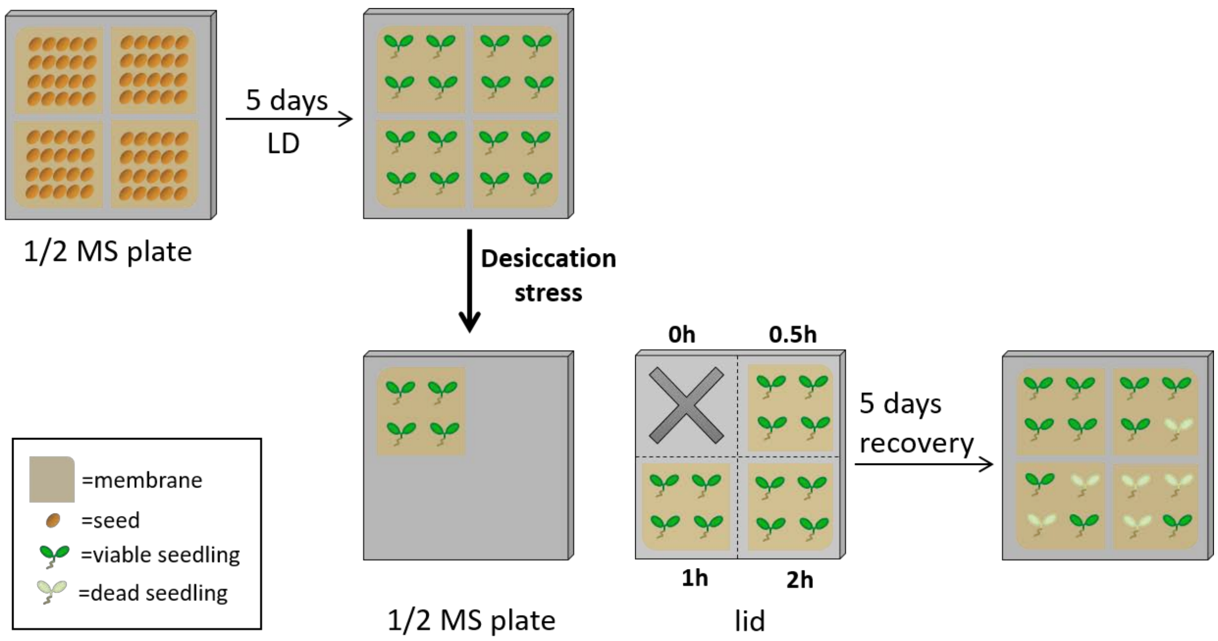

Figure S4: Desiccation stress treatment procedure.

Petri-dishes containing half-strength Murashige and Skoog medium supplemented with 0.5% sucrose (1/2 MS) were covered with Sefar Nitex membrane (03-200/54) with a pore size of 200µm/diameter. Sterile seeds were grown on top of this membrane under long day conditions (16/8 h light/dark). Desiccation stress was applied 5 days after germination. For desiccation stress, the membrane containing the young seedlings was placed in a sterile petri-dish, while constant air-flow, and transferred back on the initial ½ MS plate after 0, 0.5, 1, and 2 hours. For the 0h control the membrane was only lifted up and directly placed back on 1/2 MS to avoid possible artefacts caused solely by lifting up the membrane/seedlings. Constant airflow in a sterile bench ensured that the seedlings on the lid were exposed to desiccation stress conditions. Seedlings were grown for additional 5 days on 1/2 MS, then survival was scored.

Table S1: Oligonucleotides used for H3K27me3 and H3K4me3 ChIP qPCR.

gene name	ATG number	F	R
PI	AT5G20240	CCACATATCCTCTCCTCCATA	CCATTCCTCCTCTTTGAGAACG
SEP2	AT2G21970	TGTTTTTGATGCGTGAGGTT	CAAAGCTCTGTTGGCATCAA
SEP3	AT1G24260	GGGTTTCCAATTTTGGGTTT	GATGAATCCCATCCCCAAGT
AG	AT4G18960	TGGGTACTGAGAGGAAAGTGAGA	GGATCGTAGAAGGCAGACCA
BIP3	AT1G09080	GTGAGCTTGCGAAACGATCT	CCTCGAATCTTGCTCTCGTT
SEC31A	AT1G18830	TACAAGGAAGCAGTGGCTCA	CCCACAATTCTGTACCACCA
LTP2	AT2G38530	GCAACGGCGTTACTAACCTT	TTTAGCGGCAGATTGAAGGC
Threonyl-tRNA synthetase	AT1G17960	CTTCCGGCTTGCTTCAAACT	AGATCCCAACACCGCACTAT
UDP-Glycosyltransferase superfamily protein	AT3G55700	TTCAACCCCATGATCGAGCT	AGAAGGATCGGGGAAGTTGT
ACT7	AT5G09810	TAGTGAAAAATGGCCGATGG	CCATTCCAGTTCCATTGTCA
FUS3 (Kwon et al., 2009)	AT3G26790	GTGGCAAGTGTTGATCATGG	AGTTGGCACGTGGGAAATAG
SEP2-ATG	AT3G02310	TTTTGGGGTGAGGAAAGATG	CGCAGAGAACAGAAAGCTCA
SEP3 -ATG	AT1G24260	TGACGTTTGCAAAGAGAAGG	GCATGCTCGAACTACTGCAA
BIP3 (Song et al., 2015)	AT1G09080	CACGGTTCCAGCGTATTTCAAT	ATAAGCTATGGCAGCACCCGTT
SEC31A (Song et al., 2015)	AT1G18830	GAACTCGATTTTCAGTCCAA	TTGGATTCCATAAACCGATG

Table S2: Oligonucleotides used for qRT-PCR analysis of *BLI* and *CLF* expression.

gene name	ATG number	F	R
BLI	AT3G23980	AGAGGGAACATTTCCCTCTG	GAAACTGCTCAAGCTTACGG
ACT7	AT5G09810	CCAGGAATTGCTGACCGTAT	GGTGCAACCACCTTGATCTT
CLF	AT2G23380	TTTCGATAACCTGTTCTGCC	GTCTCCCACTACCTTTCACC
PP2A-1 (Czechowski et al., 2005)	AT1G59830	TGAGCACGCTCTTCTTGCTTTCA	GGTGGTGGCATCCATCTTGTTACA

Supplemental methods

RNA isolation and qPCR

***BLI* transcript levels:** RNA from rosette leaves was extracted using RNeasy Plant Mini Kit (Qiagen), resuspended in 30 µl RNAse-free water, and treated with DNaseI (Fermentas). cDNA was synthesized from 1 µg RNA using SuperScriptII Reverse transcriptase Kit (Invitrogen) and Oligo(dT) oligonucleotides. The obtained cDNA was diluted 1:10 and 1 µl of this dilution was used for qRT-PCR. qPCR was performed in a Chromo4 real-time PCR machine (Bio-Rad) using MESA BLUE qPCR MasterMix Plus for SYBR® Assay (Eurogentech) in a 2-step PCR program (95°C 5min, 40 x (95°C 0:15 min, 60°C 1:00 min)). Expression levels were normalized to *ACTIN7* (AT5G09810).

***CLF* transcript levels:** RNA from 14-day old seedlings was extracted using innuSPEED Plant RNA Kit (Analytik Jena), resuspended in 30 µl RNAse-free water, and treated with DNaseI (Fermentas). cDNA was synthesized from 1 µg RNA using RevertAid RT Reverse Transcription Kit (Thermo Scientific) and Oligo(dT) oligonucleotides. The obtained cDNA was diluted 1:10 and 2 µl of this dilution was used for qRT-PCR. qPCR was performed in a LightCycler 480 (Roche) using KAPA SYBR FAST qPCR Master Mix in a 2-step PCR program (95°C 5:00 min, 40 x (95°C 0:15 min, 60°C 0:30 min)). Expression levels were normalized to AT1G59830 (*PP2A-1*) (Czechowski et al., 2005).

Cloning of pGKGWG-gBLI

Genomic *BLI* (*gBLI*), containing the *BLI* coding region and 1.7 kb upstream of the transcriptional start site, was amplified from genomic DNA using oligonucleotides F: GGGGACAAGTTTGTACAAAAAAGCAGGCTGAACTGGCAATTCAGAATCGGG, R: GGGGACCACTTTGTACAAGAAAGCTGGGTGGAGAAGCTTGCTTGTCCTTCTTTTC, and introduced into pDONR201 (Invitrogen). *gBLI* was cloned into pGKGWG (Zhong et al., 2008) using GATEWAY technology (Thermo Fisher Scientific), according to the manufacturers´ instructions.

Plant transformation:

bli-1 and *bli-11* heterozygous mutants were transformed with pGKGWG-gBLI using the floral-dip method (Clough and Bent, 1998) and *Agrobacterium tumefaciens* strain GV3101 pMP90 (Koncz and Schell, 1986).

Supplemental References

Clough, S.J., and Bent, A.F. (1998). Floral dip: a simplified method for Agrobacterium-mediated transformation of Arabidopsis thaliana. Plant J *16*, 735-743.

Czechowski, T., Stitt, M., Altmann, T., Udvardi, M.K., and Scheible, W.R. (2005). Genome-wide identification and testing of superior reference genes for transcript normalization in Arabidopsis. Plant Physiol *139*, 5-17.

Koncz, C., and Schell, J. (1986). The promoter of the T L -DNA gene 5 controls the tissue-specific expression of chimeric genes carried by a novel type of Agrobacterium binary vector. Mol Gen Genet *204*, 383-396.

Kwon, C.S., Lee, D., Choi, G., and Chung, W.I. (2009). Histone occupancy-dependent and -independent removal of H3K27 trimethylation at cold-responsive genes in Arabidopsis. Plant J *60*, 112-121.

Song, Z.T., Sun, L., Lu, S.J., Tian, Y., Ding, Y., and Liu, J.X. (2015). Transcription factor interaction with COMPASS-like complex regulates histone H3K4 trimethylation for specific gene expression in plants. Proc Natl Acad Sci USA *112*, 2900-2905.

Zhong, S., Lin, Z., Fray, R.G., and Grierson, D. (2008). Improved plant transformation vectors for fluorescent protein tagging. Transgenic Res *17*, 985-989
